# Prognostic significance of the Complex "Visceral Adiposity Index" vs. simple anthropometric measures: Tehran lipid and glucose study

**DOI:** 10.1186/1475-2840-11-20

**Published:** 2012-03-07

**Authors:** Bozorgmanesh Mohammadreza, Hadaegh Farzad, Khalili Davoud, Azizi Fereidoun Prof

**Affiliations:** 1 Prevention of Metabolic Disorders Research Center, Research Institute for Endocrine Sciences (RIES), Shahid Beheshti University of Medical Sciences, Tehran, Iran; 2 Endocrine Research Center, Research Institute for Endocrine Sciences (RIES), Shahid Beheshti University of Medical Sciences, P.O. Box 19395-4763, Tehran, Islamic Republic of Iran

**Keywords:** Body mass index, Cardiovascular disease, Prediction, Visceral adiposity index, Waist- to-height ratio, Waist-to-hip ratio

## Abstract

**Background:**

Visceral adiposity index (VAI) has recently been suggested to be used as a surrogate of visceral adiposity. We examined if VAI could improve predictive performances for CVD of the Framingham's general CVD algorithm (a multivariate model incorporating established CVD risk factors). We compared the predictive abilities of the VAI with those of simple anthropometric measures i.e. BMI, waist-to-height ratio (WHtR) or waist-to-hip ratio (WHpR).

**Design and methods:**

In a nine-year population-based follow-up, 6 407 (2 778 men) participants, free of CVD at baseline, aged ≥ 30 years were eligible for the current analysis. The risk of CVD was estimated by incorporating VAI, BMI, WHpR, and WHtR, one at a time, into multivariate accelerated failure time models.

**Results:**

We documented 534 CVD events with the annual incidence rate (95%CIs) being 7.3 (6.4-8.3) among women and 13.0 (11.7-14.6) among men. Risk of future CVD increased with increasing levels of VAI among both men and women. VAI was associated with multivariate-adjusted increased risk of incident CVD among women. However, the magnitude of risk conferred by VAI was not significantly higher than those conferred by BMI, WHpR, or WHtR. Among men, after adjustment for established CVD risk factors, VAI was no longer associated with increased risk of CVD. VAI failed to add to the predictive ability of the Framingham general CVD algorithm.

**Conclusions:**

Using VAI instead of simple anthropometric measures may lead to loss of much information needed for predicting incident CVD.

## Introduction

There is no consensus on the definition of obesity or on specific aspects of obesity that contribute to the risk of CVD [1]. The precise measurement of the total amount of body fat and its regional distribution is possible by using computed tomography (CT), dual-energy X-ray absorption [2]. Magnetic resonance imaging (MRI), like CT, can separate visceral fat from subcutaneous fat and since there is no radiation involved, it can perform a total body scan for maximal accuracy and fat distribution. However, these methods are primarily used at the research level. Besides, they are time-consuming, costly, and not routinely available. Accordingly there is a need for simple techniques that can discriminate regional fat. Amato et al. have recently individuated a novel sex-specific index based on waist circumference, body mass index (BMI), triglycerides (TGs), high-density lipoprotein cholesterol (HDL-C), and indirectly expressing visceral fat [3] and termed it the visceral adiposity index (VAI). VAI had significant correlation with visceral adiposity and its increase was strongly associated with cardiometabolic risk. However, the prospective relation between VAI and CVD is less clear [3]. Clinical importance of visceral adiposity lies in its association with health risks like CVD. Therefore, from clinical point-of-view, indices developed to measure visceral adiposity should be examined with respect to their ability to predict risks known to be associated with it [4,5].

Using data from a large community-based study, we examined if VAI would improve CVD prediction currently made by multivariate algorithms and if VAI could add to the predictive ability of the simple anthropometric measures of adiposity i.e. BMI, waist-to-height ratio (WHtR) or waist-to-hip ratio (WHpR).

## Methods

### Study population

Detailed descriptions of the Tehran lipid and glucose study (TLGS) have been reported elsewhere [6]; in brief, the TLGS is a large scale, long term, community-based prospective study performed on a representative sample of residents of district 13 of Tehran, the capital of Iran. The TLGS has two major components: a cross-sectional prevalence study of noncommunicable disease and associated risk factors, implemented between March 1999 and December 2001, and a prospective follow-up study. Data collection is ongoing, designed to continue for at least 20 years, at 3-year intervals. Participants were categorized into the cohort (n = 9375) and intervention groups (n = 5630), the latter to be educated for implementation of life style modifications. For the current study, among participants aged ≥ 30 (n = 8,071), we selected those who participated in the follow-up study until 20 March 2009 (n = 7,154). After exclusions (344 prevalent CVD and 382 missing data), 6,407 (2,778 men) participants remained eligible (response rate 95%), contributing to a 54,950 person-year follow up. At the time of this study, the median follow up time was 9.1 years. Participants were provided with information regarding the results of their examinations and were given appropriate medical advice.

### Clinical and laboratory measurements

Using a pretested questionnaire, a trained interviewer collected information on demographic data, family history of premature CVD, past medical history of CVD, and smoking status. Detailed description of clinical and laboratory measurements has been provided in appendices. Weight was measured, with subjects minimally clothed without shoes, using digital scales (Seca 707: range 0.1-150 kg) and recorded to the nearest 100 g. Height was measured in a standing position without shoes, using tape meter while shoulders were in a normal alignment. Waist circumference (WC) was measured at the umbilical level and that of the hip at the maximum level over light clothing, using an unstretched tape meter, without any pressure to body surface and measurements were recorded to the nearest 0.1 cm [7]. BMI (kg.m^-2^) was calculated as weight (kg) divided by square of the height (m^2^). WHpR was calculated as WC (cm) divided by hip circumference (cm) and WHtR was calculated as WC divided by height (cm). After a 15-minute rest in the sitting position, two measurements of blood pressure were taken, on the right arm, using a standardized mercury sphygmomanometer (calibrated by the Iranian Institute of Standards and Industrial Researches); the mean of the two measurements was considered as the participant's blood pressure.

A blood sample was drawn between 7:00 and 9:00 AM from all study participants, after 12 to 14 hours overnight fasting. All the blood analyses were undertaken at the TLGS research laboratory on the day of blood collection. Plasma glucose was measured using an enzymatic colorimetric method with glucose oxidase. Fasting plasma glucose (FPG) measurement was performed for all participants, and the standard 2-hour post-challenge plasma glucose (2 h-PCPG) test for those not on glucose-lowering drugs. Total cholesterol (TC) was assayed, using the enzymatic colorimetric method with cholesterol esterase and cholesterol oxidase. High-density lipoprotein cholesterol (HDL-C) was measured after precipitation of the apolipoprotein B containing lipoproteins with phosphotungistic acid. TGs were assayed using enzymatic colorimetric assay with glycerol phosphate oxidase. Analyses were performed using Pars Azmon kits (Pars Azmon Inc., Tehran, Iran) and a Selectra 2 auto-analyzer (Vital Scientific, Spankeren, Netherlands). All samples were analyzed when internal quality control met the acceptable criteria. The intra and inter-assay coefficients of variation were both < 2.2% for plasma glucose, and 0.5 and 2% for TC, respectively [6].

### Outcome measurements

Details of cardiovascular outcomes have been published elsewhere [8]. In this ongoing study every TLGS' participant is followed up for any medical event during the previous year, by telephone. They are questioned by a trained nurse regarding any medical conditions or whether a related event have occurred, a trained physician collects complementary data during a home visit and a visit to the respective hospital to collect data from the participants medical files. In the case of mortality, data are collected from the hospital or the death certificate by an authorized local physician. Collected data are evaluated by an outcome committee consisting of a principal investigator, an internist, an endocrinologist, a cardiologist, an epidemiologist, and the physician who collects the outcome data. Other experts are invited for evaluation of non-communicable disorders, as needed. A specific outcome for each event is assigned according to International Statistical Classification of Diseases and Related Health Problems criteria (10th Revision), and the American Heart Association classification for cardiovascular events [6,9,10]. Coronary heart disease (CHD) includes cases of definite myocardial infarction (MI) diagnosed by electrocardiogram (ECG) and biomarkers, probable MI (positive ECG findings plus cardiac symptoms or signs and biomarkers showing negative or equivocal results), unstable angina pectoris (new cardiac symptoms or changing symptom patterns and positive ECG findings with normal biomarkers), angiographic proven CHD and CHD death. CVD is specified as a composite measure of any CHD events, stroke, or cerebrovascular death.

### Definition of terms

Following Amato et al. [3] we defined VAI as:

$$\begin{array}{c} \text{Males}=\left(\frac{\text{WC}}{39.68+1.88\times \text{BMI}}\right)\times \left(\frac{\text{TGs}}{1.03}\right)\times \left(\frac{1.31}{\text{HDL - C}}\right)\\ \text{Females}=\left(\frac{\text{WC}}{36.58+1.89\times \text{BMI}}\right)\times \left(\frac{\text{TGs}}{0.81}\right)\times \left(\frac{1.52}{\text{HDL - C}}\right) \end{array}$$

assuming VAI = 1 in healthy non-obese subjects with normal adipose distribution and normal TG and HDL levels. A previous history of CVD reflected any prior diagnosis of CVD by a physician. A current smoker was defined as a person who smokes cigarettes daily or occasionally. Participants using oral hypoglycemic agents or insulin were considered as having diabetes. Diabetes was also ascertained in participants with FPG ≥ 7.0 mmol.l^-1 ^or 2 h-PCPG ≥ 11.1 mmol.l^-1 ^[11]. Non-HDL-C was calculated by subtracting HDL-C from total cholesterol. For each participant, free of CVD at baseline, the baseline risk of CVD was calculated by re-estimating the Framingham's "general CVD risk prediction algorithm [12].

### Statistics analysis

Findings on covariate variables are expressed as means (SD) or percentages for continuously distributed and categorical variables, respectively. We tested for trends across VAI quintiles by using the median in each quartile as a predictor, separately for each sex. Statistical significance in trends was examined by implementing General Linear Models. The Log-Rank test and Cox test were used to examine the significance of trends in incident rates and survivor functions.

In the analysis of CVD outcome, VAI, BMI, WHpR and WHtR were assessed using accelerated failure time method: Weibull survival regression model. Survival time was the time from start of the follow-up period to the date of the first incident, CVD event (failure). The censoring time of an individual was the time from entry into the study to loss to follow-up or the end of the study, whichever happened first. Censored observation meant the individuals either refused to participate further in the study (lost to follow-up), died (from none-CVD causes), when death was not the study outcome (competing risk) or continued until the study was ended (administrative censoring). Valid comparison of hazards ratios (HRs) for different continuous measures requires that the units of both variables to be comparable. We, thus, estimated sex-specific age-adjusted hazard ratios (HRs), with 95% confidence intervals (CI) for CVD events for a one-SD increment in VAI and each respective anthropometric parameter. We controlled our regression analyses for cofounding bias due to potential confounders i.e. age, systolic blood pressure, using antihypertensive drugs, total and HDL cholesterol, diabetes and smoking [13].

We compared predictive performance of the VAI with those of the studied anthropometric variables in terms of the effect size (HR), calibration and discrimination, added predictive ability, and explained variation.

Wald tests of the linear hypotheses concerning the Weibull survival regression models coefficients (paired homogeneity test) were performed to test the null hypotheses that the hazard ratios (effect size) for VAI were equal to those for anthropometric measures. We assessed collinearity of BMI, WHpR, and WHtR, with VAI using variance inflation factor (VIF). VIFs > 10 warrant caution [14].

Calibration, as it is phrased in reference [15] describes how closely predicted probabilities agree numerically with actual outcomes [16,17]. A test very similar to the Hosmer-Lemeshow test has been proposed by Nam and D'Agostino. We calculated the NamD'Agostino *χ^2 ^*to examine calibration for prediction models [15]. As suggested by D'Agostino and Nam, calibration chi-square values greater than 20 (P < 0.01) suggest lack of adequate calibration [15].

Discrimination is the ability of a prediction model to separate those who develop incident CVD events from those who do not and is quantified by the Harrell's *C *statistic [18]. In the survival analysis, *C *statistic [19] measures the probability that a randomly selected person who developed an event, at the certain specific time has a higher risk score than a randomly selected person who did not develop an event during the same, specific follow-up interval [20].

Discriminations measures are not sensitive to changes in absolute risk [21]. We, thus, calculated absolute and relative integrated discrimination improvement index *(*IDI) and cut-point-based and cut-point-free net reclassification improvement index (NRI). IDI and NRI are measures of predictive ability added to an old model by a newer one [21]. Bootstrapping method was implemented in order to obtain bias-corrected 95% confidence intervals (95% CIs).

We hereby certify that all applicable institutional and governmental regulations concerning the ethical use of human volunteers were followed during this research. Informed written consent was obtained from all participants and the Ethical Committee of Research Institute for Endocrine Sciences approved this study.

The statistical significance level was set at a two-tailed type I error of 0.05. All statistics analyses were performed using STATA version 12 (STATA, College Station, Texas USA) and SAS 9.2 (SAS Institute Inc., Cary, NC, USA).

## Results

For a median follow up of 9 years, 6,407 (3,629 women) adult participants of the TLGS contributed to a total of 54,950 person-years follow up. We documented 534 CVD events with the annual incidence rate of CVD events being 9.7 (95% 8.9-10.6) per 1000 person: women 7.3 (6.4-8.3) and men 13.0 (11.7-14.6).

**Figure 1:**
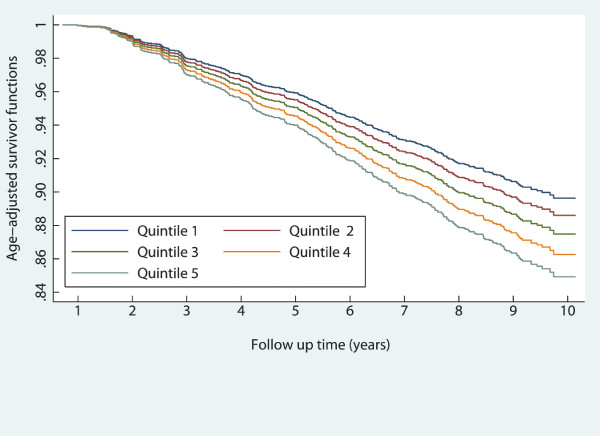
**Age-adjusted survival curves across quintiles of the visceral adiposity index: men**.

**Figure 2:**
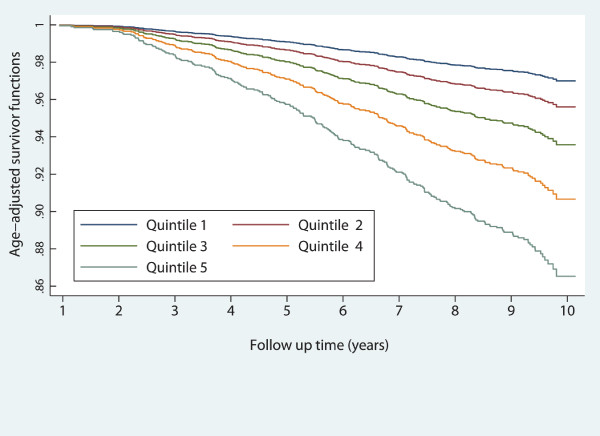
**Age-adjusted survival curves across quintiles of the visceral adiposity index: women**.

Participants' characteristics are shown according to baseline VAI quintiles in Tables 1 and 2. In general, CVD risk factors levels at baseline increased in stepwise fashion across VAI quintiles; except for smoking. The annual incidence rate of CVD events showed an increasing trend across quintiles of VAI among women (P < 0.001). Age-adjusted survival functions for quintiles of VAI have been compared in Figures 1 and 2. Age-adjusted CVD-free survival probability decreased significantly across VAI quintiles among both men and women (P values < 0.001).

**Table 1:** Basal characteristics of participants across VAI quintiles, among men

	Q1	Q2	Q3	Q4	Q5	
	
	N = 658	N = 563	N = 614	N = 475	N = 464	P for trend
	
VAI range	0.27-1.49	1.49-2.26	2.26-3.24	3.24-4.88	4.89-29.84	
Age (years)	41.43 (10.86)	45.18 (11.36)	47.70 (11.80)	48.30 (11.64)	49.79 (11.11)	0.010
Life style modification	263 (0.4)	220 (0.39)	221 (0.36)	185 (0.39)	190 (0.41)	0.764
Smoking	184 (0.28)	157 (0.28)	177 (0.29)	132 (0.28)	144 (0.31)	0.451
Diabetes	32 (0.05)	44 (0.08)	66 (0.11)	89 (0.19)	100 (0.22)	< 0.001
Anti-hypertensive drug	46 (0.07)	39 (0.07)	43 (0.07)	38 (0.08)	32 (0.07)	0.720
SBP (mm Hg)	113.73 (16.81)	119.67 (18.71)	123.54 (20.84)	126.18 (21.21)	129.35 (21.43)	< 0.001
DBP (mm Hg)	74.98 (9.81)	78.03 (10.26)	80.16 (10.90)	81.35 (10.73)	82.83 (10.62)	< 0.001
Weight (kg)	64.06 (10.95)	67.41 (12.08)	69.62 (11.33)	71.27 (12.43)	72.12 (11.18)	< 0.001
Waist (cm)	82.01 (10.83)	87.93 (11.67)	91.12 (10.95)	93.68 (11.31)	96.31 (10.17)	< 0.001
Height (cm)	156.46 (5.78)	156.13 (6.11)	155.25 (5.69)	155.54 (5.92)	155.27 (5.76)	< 0.001
Hip circumference (cm)	102.24 (8.78)	104.40 (9.34)	106.00 (9.59)	106.77 (9.50)	106.82 (9.14)	< 0.001
BMI (kg.m^-2^)	26.20 (4.50)	27.66 (4.73)	28.90 (4.58)	29.42 (4.63)	29.90 (4.25)	< 0.001
WHpR	80.16 (7.37)	84.20 (7.97)	86.01 (7.52)	87.77 (7.74)	90.30 (7.50)	< 0.001
WHtR	52.51 (7.40)	56.42 (7.93)	58.77 (7.43)	60.29 (7.43)	62.10 (6.81)	< 0.001
TC (mmol.l^-1^)	5.07 (0.96)	5.39 (1.03)	5.76 (1.07)	5.97 (1.15)	6.30 (1.35)	< 0.001
HDL-C (mmol.l^-1^)	1.43 (0.27)	1.28 (0.25)	1.16 (0.21)	1.06 (0.19)	0.91 (0.18)	< 0.001
TGs (mmol.l^-1^)	0.86 (0.21)	1.28 (0.28)	1.70 (0.34)	2.19 (0.44)	3.59 (1.38)	< 0.001
Ln-TGs	-0.19 (0.26)	0.23 (0.21)	0.51 (0.20)	0.76 (0.20)	1.22 (0.32)	< 0.001
FPG (mmol.l^-1^)	5.01 (1.17)	5.26 (1.55)	5.53 (2.00)	5.91 (2.46)	6.23 (2.59)	< 0.001
PCPG (mmol.l^-1^)	5.79 (1.76)	6.25 (2.16)	6.94 (3.18)	7.56 (3.35)	8.35 (3.90)	< 0.001
Incident CVD	9.7 (7.4-12.6)	13.9 (10.9-17.7)	12.3 (9.7-15.8)	15.3 (11.9-19.7)	15.2 (11.8-19.7)	0.076

BMI, body mass index; CVD, cardiovascular disease; DBP, diastolic blood pressure; FPG, fasting plasma glucose; HDL-C, high-density lipoprotein cholesterol; MAP, mean arterial pressure; PCPG, 2-hour post-challenge plasma glucose; SBP, systolic blood pressure; TC, total cholesterol; TGs, triglycerides; VAI, visceral adiposity index; WHpR, waist-to-hip ratio; WHtR, waist-to-height ratio

**Table 2:** Basal characteristics of participants across VAI quintiles, among women

	Q1	Q2	Q3	Q4	Q5	
	
	N = 654	N = 718	N = 677	N = 793	N = 783	P for trend
	
VAI range	0.26-1.49	1.49-2.25	2.26-3.24	3.24-4.87	4.88-46.74	
Age (years)	49.11 (14.46)	48.5 (13.32)	48.27 (12.78)	48.46 (12.55)	46.97 (11.87)	< 0.001
Life style modification	250 (0.38)	285 (0.40)	256 (0.38)	315 (0.40)	303 (0.39)	0.874
Smoking	26 (0.04)	21 (0.03)	32 (0.05)	26 (0.03)	28 (0.04)	0.846
Diabetes	22 (0.03)	47 (0.07)	86 (0.13)	150 (0.20)	202 (0.27)	< 0.001
Anti-hypertensive drug	53 (0.08)	101 (0.14)	121 (0.18)	151 (0.19)	197 (0.25)	< 0.001
Lipid lowering drug	7 (0.01)	11 (0.02)	16 (0.02)	44 (0.06)	98 (0.13)	< 0.001
SBP (mm Hg)	120.28 (20.67)	121.48 (21.20)	123.49 (19.52)	123.22 (16.87)	124.92 (18.51)	< 0.001
DBP (mm Hg)	75.91 (11.83)	77.66 (11.57)	79.79 (11.60)	80.03 (10.27)	80.73 (11.31)	< 0.001
Weight (kg)	67.94 (11.85)	73.70 (11.28)	76.37 (11.45)	78.47 (11.72)	80.30 (11.53)	< 0.001
Waist (cm)	82.91 (10.83)	88.91 (10.01)	91.64 (9.58)	93.85 (9.29)	95.69 (8.85)	< 0.001
Height (cm)	168.79 (7.05)	169.09 (6.48)	169.11 (6.48)	168.74 (5.93)	169.34 (6.70)	< 0.001
Hip circumference (cm)	93.12 (6.73)	96.22 (6.62)	97.41 (6.06)	98.36 (6.67)	99.05 (6.50)	< 0.001
BMI (kg.m^-2^)	23.84 (3.85)	25.79 (3.79)	26.67 (3.45)	27.52 (3.54)	27.97 (3.39)	< 0.001
WHpR	88.81 (7.17)	92.26 (6.50)	93.96 (6.28)	95.35 (6.01)	96.59 (6.20)	< 0.001
WHtR	49.20 (6.67)	52.67 (6.41)	54.24 (5.77)	55.65 (5.43)	56.56 5.37 ()	< 0.001
TC (mmol.l^-1^)	4.94 (0.98)	5.36 (0.99)	5.50 (1.01)	5.63 (1.07)	5.87 (1.15)	< 0.001
HDL-C (mmol.l^-1^)	1.22 (0.26)	1.04 (0.18)	0.95 (0.17)	0.87 (0.16)	0.77 (0.15)	< 0.001
TGs (mmol.l^-1^)	0.98 (0.26)	1.51 (0.28)	2.01 (0.39)	2.62 (0.50)	4.35 (1.63)	< 0.001
Ln-TGs	-0.06 (0.28)	0.39 (0.19)	0.68 (0.19)	0.95 (0.19)	1.41 (0.32)	< 0.001
FPG (mmol.l^-1^)	5.26 (1.44)	5.26 (1.17)	5.47 (1.64)	5.80 (1.98)	6.07 (2.34)	< 0.001
PCPG (mmol.l^-1^)	5.80 (2.79)	6.08 (2.77)	6.61 (3.18)	7.09 (4.01)	7.66 (4.01)	< 0.001
Incident CVD	2.4 (1.4-4.1)	3.9 (2.7-5.8)	6.3 (4.5-8.7)	10.7 (8.5-13.5)	11.9 (9.5-14.8)	< 0.001

BMI, body mass index; CVD, cardiovascular disease; DBP, diastolic blood pressure; FPG, fasting plasma glucose; HDL-C, high-density lipoprotein cholesterol; MAP, mean arterial pressure; PCPG, 2-hour post-challenge plasma glucose; SBP, systolic blood pressure; TC, total cholesterol; TGs, triglycerides; VAI, visceral adiposity index; WHpR, waist-to-hip ratio; WHtR, waist-to-height ratio

Risk of future CVD increased with increasing levels of VAI among both men and women. Among women, but not men, the increased risk resisted adjustments for the CVD risk factors (Table 3). As shown in Table 3, HRs for CVD of WHpR and WHtR were consistently higher than those of VAI among both men and women. These superiorities, however, did not reach statistical significance.

**Table 3:** Hazard ratios for incident CVD of VAI vs. WHpR and WHtR

			HR 95% CIs	**vs. VAI**^**a**^
	Men	VAI	1.18 (1.07-1.30)	-
		WHpR	1.37 (1.22-1.55)	0.061
		WHtR	1.38 (1.21-1.58)	0.068
		BMI	1.28 (1.13-1.45)	0.355
Age-adjusted	Women	VAI	1.27 (1.18-1.37)	-
		WHpR	1.52 (1.31-1.76)	0.133
		WHtR	1.45 (1.27-1.66)	0.229
		BMI	1.26 (1.12-1.43)	0.682
	Men	VAI	1.05 (0.94-1.18)	-
		WHpR	1.23 (1.07-1.40)	0.078
		WHtR	1.21 (1.05-1.39)	0.123
		BMI	1.08 (0.95-1.23)	0.301
Multivariate-adjusted^b, c^	Women	VAI	1.17 (1.07-1.28)	-
		WHpR	1.42 (1.22-1.65)	0.078
		WHtR	1.36 (1.18-1.56)	0.146
		BMI	1.04 (1.01-1.07)	0.352

CVD, cardiovascular disease; HR, hazard ratio; VAI, visceral adiposity index; WHpR, waist-to-hip ratio; WHtR, waist-to-height ratio

a. *P *values were derived from Wald tests of the linear hypotheses concerning the Weibull regression models coefficients (paired homogeneity test). As such, we tested the null hypotheses that the hazard ratios (effect size) for VAI were equal to those for WHpR, WHtR, or BMI

b. Adjusted for the effects of age, systolic blood pressure, anti-hypertensive medication use, total and high-density lipoprotein cholesterol, diabetes, and smoking

c. To avoid over-adjustment, VAI was not adjusted for high-density lipoprotein cholesterol since it was a component of VAI

We observed that among men, 0.7% (95% CIs 0.09-1.9%) of variations in CVD-free survival time was explained VAI. The corresponding figures were 4.5% (95% CIs 2.4-7.2%) for WHtR, 6.1 (95% CIs 3.6-9.0%) for WHpR, and 0.8% (95% CIs 0.1-2.2%) for BMI. Among women, 5.0% (95% CIs 2.7-7.8%) of variations in CVD-free survival time was explained VAI. The corresponding figures were 7.3% (95% CIs 4.5-10.7%) for WHtR, 10.0 (95% CIs 6.6-13.9%) for WHpR, and 1.4% (95% CIs 0.4-3.0%) for BMI.

Harrell's *C *(95% CIs) and Nam-D'Agostino *χ*^2 ^(P for lack of fit) for the CVD risk based on the Framingham general CVD algorithm were 0.777 (0.754-0.802) and 15.8 (0.070) among men; the corresponding figures were 0.778 (0.704-0.802) and 13.6 (0.135) for VAI, 0.712 (0.700-0.729) and 12.2 (0.200) for BMI, 0.778 (0.704-0.802) and 17.0 (< 0.0.048) for WHtR, and 0.778 (0.704-0.802) and 14.6 (0.103) for WHpR. The Harrell's *C *(95% CIs) and NamD'Agostino *χ*^2 ^(P for lack of fit) for the CVD risk based on the Framingham general CVD algorithm were 0.841 (0.820-0.862) and 13.9 (0.126) among women. The corresponding figures were 0.841 (0.820-0.862) and 11.1 (0.266) for VAI, 0.835 (0.814-0.858) and 35.9 (< 0.001) for BMI, 0.838 (0.816-0.859) and 42.0 (< 0.0001) for WHtR, and 0.839 (0.8170.860) and 23.2 (0.005) for WHpR.

As shown in Table 4, VAI failed to add to the predictive ability of the Framingham general CVD algorithm. Moreover, among women, when VAI was directly compared to the WHtR, WHpR, and BMI, NRI and IDI statistics were negative, indicating that WHpR, WHtR, and BMI predicted CVD better than did VAI. The only exception insinuated to the findings was that, among men, VAI outperformed BMI as denoted by all indices of added predictive abilities.

**Table 4:** Added predictive ability conferred by VAI. male

		Women				Men		
		**95% CIs**		**P-value**	**Statistic**	**95% CIs**		**P-value**

General CVD risk^b^								
Absolute IDI (%)	-0.005	-0.009	-0.002	0.004	-0.003	-0.006	-0.001	0.006
Relative IDI (%)	-0.039	-0.064	-0.014	0.002	-0.025	-0.042	-0.007	0.005
Cut-point-based NRI ^c ^(%)	-0.018	-0.053	0.017	0.310	-0.004	-0.028	0.021	0.764
Cut-point-free NRI (%)	-0.221	-0.354	-0.089	0.001	-0.173	-0.258	-0.089	0.000
WHtR								
Absolute IDI (%)	-0.044	-0.054	-0.035	0.000	-0.017	-0.022	-0.012	0.000
Relative IDI (%)	-0.262	-0.298	-0.225	0.000	-0.115	-0.147	-0.084	0.000
Cut-point-based NRI ^c ^(%)	-0.077	-0.141	-0.013	0.019	-0.008	-0.042	0.025	0.632
Cut-point-free NRI (%)	-0.052	-0.140	0.036	0.250	0.010	-0.070	0.091	0.800
WHpR								
Absolute IDI (%)	-0.028	-0.037	-0.020	0.000	-0.012	-0.017	-0.008	0.000
Relative IDI (%)	-0.185	-0.228	-0.142	0.000	-0.085	-0.113	-0.057	0.000
Cut-point-based NRI ^c ^(%)	-0.027	-0.084	0.030	0.345	0.003	-0.030	0.035	0.878
Cut-point-free NRI (%)	-0.177	-0.294	-0.060	0.003	-0.122	-0.207	-0.037	0.005
BMI								
Absolute IDI (%)	-0.038	-0.047	-0.029	0.000	0.055	0.042	0.068	0.000
Relative IDI (%)	-0.233	-0.270	-0.197	0.000	0.727	0.481	0.973	0.000
Cut-point-based NRI ^c ^(%)	-0.067	-0.125	-0.010	0.022	0.346	0.286	0.406	0.000
Cut-point-free NRI (%)	-0.031	-0.120	0.059	0.501	0.507	0.423	0.592	0.000

BMI, body mass index; CVD, cardiovascular disease; IDI, integrated discrimination improvement index; NRI, net reclassification improvement index; VAI, visceral adiposity index; WHpR, waist-to-hip ratio; WHtR, waist-to-height ratio

a. Negative signs indicate less predictive ability for VAI as compared to the general CVD algorithm, WHtR, WHpR, or BMI

b. Calculated in accordance with D'Agostino, R.B., Sr., et al., *General Cardiovascular Risk Profile for Use in Primary Care: The Framingham Heart Study*. Circulation, 2008. **117**(6): p. 743-753

For cut-point based NRI, the cut-points were set at 0.05, 0.1, and 0.2 of estimated risk

**Figure 3:**
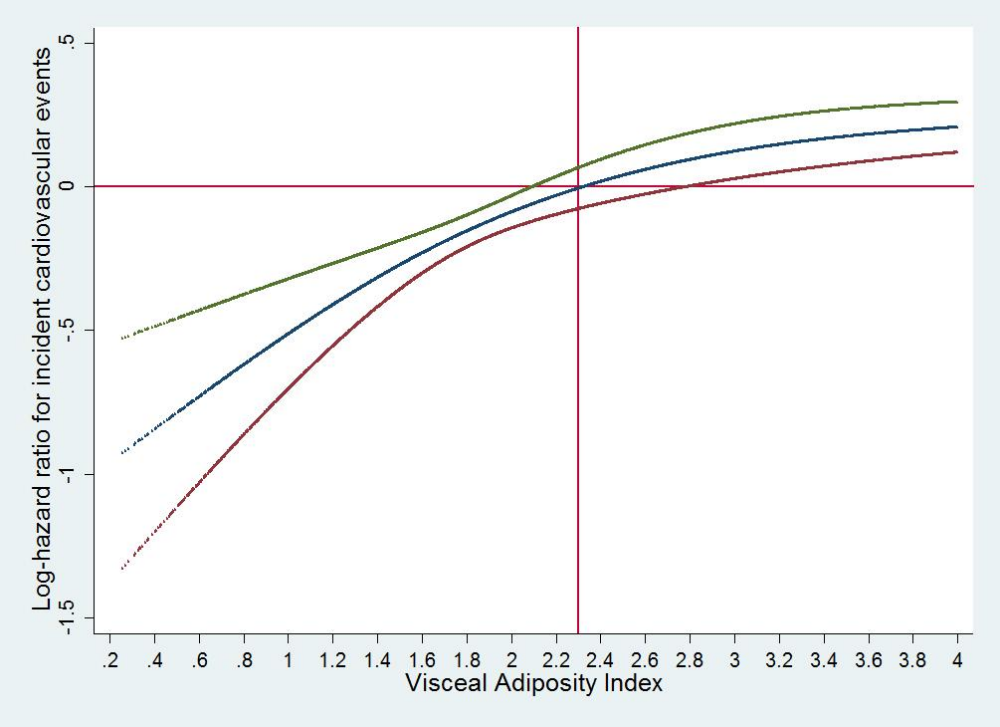
**Non-linear contribution of the visceral adiposity index to the risk of incident cardiovascular disease**.

Multivariate restricted cubic splines regression analysis demonstrated that VAI-CVD dose- response relations had no threshold and yielded straight lines when risk of disease was plotted on a logarithmic scale (Figure 3). The incident CVD risk corresponding to VAI = 2.3 was null; above this value VAI conferred hazard for incident CVD in a linear fashion. VAI values below 2.3 appeared to provide some protection against CVD.

HRs for incident CVD of lifestyle modification intervention measures was 0.89 (95% CIs 0.70-1.12, P value = 0.324) among men and 1.11 (95% 0.85-1.45, P value = 0.435) among women. Intervention measures did not contribute to the risk of CVD; neither did the intervention measures modify the effects of VAI on the risk of incident CVD (P for interaction: men 0.304 and women 0.711).

VIFs were all < 10 and therefore collinearity did not appear to be a problem.

## Discussion

In this prospective cohort of men and women, we found associations between higher VAI and risk of incident CVD as compared to simple, commonly available anthropometric measures. This community-based study demonstrated that a 1-SD increase in VAI carries 18-27% increase in age-adjusted risk of future CVD. After adjustment for traditional CVD risk factors however, it was only among women that VAI retained its predictability for incident CVD events. VAI failed to add to the predictive ability of the Framingham's "general CVD algorithm," neither it did so to those of BMI, WHpR, or WHtR. The interesting finding of our study was that using VAI instead of simple anthropometric measures of adiposity may lead to loss of considerable information needed for predicting incident CVD. WHtR and WHpR explained greater part of variations in the CVD-free survival time than VAI did.

Establishing the shape of associations between risk factors and CVD is important to gauge the potential for prevention [22,23]. We observed that dose-response association between VAI and CVD risk had no threshold. The incident CVD risk corresponding to the VAI = 2.3 was null. However, the slope of the dose-response association was steeper for VAI values smaller than 2.3 than those greater than 2.3. VAI values below 2.3 appeared to provide some protection against CVD. Above this value VAI conferred hazard for incident CVD in a linear fashion. As such, irrespective of the level of the VAI, a given increase in VAI levels above 2.3 would be accompanied by the same proportional increase in risk of incident CVD regardless of the initial risk. Meanwhile, a given decrease in VAI levels below 2.3 would be accompanied by the same proportional reduction in risk of incident CVD regardless of the initial risk. Our findings support previous reports showing the best cut-off point for VAI to be around 2.2 [24].

Adjusting for potential intermediates in the association between adiposity and CVD generally attenuates relative risks for the various indices [25,26]. We observed that after adjustment for CVD risk factors, men's VAI levels were no longer associated with risk of incident CVD. Moreover, VAI did not improve the predictive ability of the Framingham general CVD risk prediction rule; WHtR and in particular WHpR were both superior to VAI in predicting risk of incident CVD, particularly among women. We also estimated the improvements across different categories of risk of the Framingham risk algorithm and observed negligible improvements. Only one percent of participants with moderate CVD risk were correctly reclassified (data are available upon the request). It has been shown that measures of adiposity are correlated with cardiovascular risk although no single adiposity measure can be identified as the best predictor [27]. In line with our findings, however, WHtR and WHpR have been shown to be superior to BMI [28-31]. Across quintiles of VAI, contribution of WHtR and WHpR to risk of incident CVD remained essentially the same (data available upon the request from authors); indicating that VAI did not modify effects of these risk factors on the risk of CVD. The finding that VAI was not better than WHpR or WHtR, at least in part, could possibly be explained by high correlation between WHpR and WHtR and components of VAI i.e. BMI, waist circumference, HDL-C, and TGs [32]. Anthropometric measures were previously demonstrated to keep pace with a combination of TGs and waist circumference, "lipid accumulation product," in predicting incident CVD [5]. Technological developments including computer tomography scans and MRI, made it possible to precisely measure specific adipose tissue depots such as visceral adipose tissue mass [33]. We are aware of one prospective study in which visceral adiposity mass has directly been measured and compared to its anthropometric indices with respect to the CVD prediction [34]. Interestingly, clinical measurements of abdominal obesity were reported to be better predictors of CVD progression than tomography assessment in women [34]. These findings underscore the fact that there is so much room for investigations on adiposity measures that could improve CVD prediction above and beyond what have already been achieved by simple anthropometric measures.

The hypothesis that visceral adiposity would explain more variance in CVD risk factors than general adiposity was not supported in a relatively large sample of black and white adolescents [35].

"Is the visceral adiposity worth the trouble or expenses involved in accurate measurements for prediction of future CVD?" While current evidences point to the importance of using clinical measurements of abdominal obesity to identify individuals at increased risk for atherosclerosis [34], evidences are not firm enough to recommend precise measurement of the visceral adiposity mass for predicting incident CVD [27]. Moreover, Moebus et al. have demonstrated that the importance of different combinations of metabolic syndrome changes with age and between sexes putting emphasis on a tailored approach towards very young or very old subjects [36]. Sex- and age-specific VAI, thus, may confer stronger predictive capacity.

Strengths of the present study lie in its prospective nature, the use of a large population-based-cohort of both sexes, accurate and valid data on risk factors at baseline, continuous surveillance of mortality and CVD events based on standard criteria.

Some limitations to our study merit mentioning. First, in this study, no data was available about TGs lowering drugs. Second, the population studied was of Persian ancestry, our results, thus, cannot be readily extrapolated to other populations. The debate over how best to define visceral adiposity is complicated by observations suggesting that surrogates of adiposity may each perform better in predicting CVD risk in specific populations, depending on sex, age, and ethnicity [1].

## Conclusion

We demonstrated that the VAI was independently associated with an increased risk of incident CVD among women and that the magnitude of this risk due to VAI was not significantly higher than those due to BMI, WHpR, or WHtR. However, among men, after controlling common CVD risk factors, we observed that VAI was not associated with any significant increased risk of incident CVD. The increased risk observed among women, however, resisted all adjustment. Using VAI instead of simple anthropometric measures of adiposity may lead to loss of considerable information needed for predicting incident CVD.

## Abbreviations

BMI: Body mass index; CHD: Coronary heart disease; CVD: Cardiovascular disease; CT: By computed tomography; ECG: Electrocardiography: HDL-C: High-density lipoprotein cholesterol; MI: Myocardial infarction; MRI: Magnetic resonance imaging; TGs: triglycerides; TLGS: Tehran lipid and glucose study; VAIV: isceral adiposity index; VIF: Variance inflation factor; WC: Waist circumference; WHpR: Waist-to-hip ratio; WHtR: Waist-to-height ratio.

## Competing interests

The authors declare that they have no competing interests.

## Authors' contributions

MR conceptualized and designed the study, prepared and analyzed data, interpreted the results obtained, and drafted the manuscript. FH, DK, and FA reviewed/edited the manuscript. All authors gave their final approval of the version to be published. All authors read and approved the final manuscript

## References

[B1] Litwin SE Which Measures of Obesity Best Predict Cardiovascular Risk? J Am Coll Cardiol 2008 52 616 619 10.1016/j.jacc.2008.05.017 18702963

[B2] Flegal KM Shepherd JA Looker AC Graubard BI Borrud LG Ogden CL Harris TB Everhart JE Schenker N Comparisons of percentage body fat, body mass index, waist circumference, and waist-stature ratio in adults American Journal of Clinical Nutrition 2009 89 500 10.3945/ajcn.2008.26847 19116329 PMC2647766

[B3] Amato MC Giordano C Galia M Criscimanna A Vitabile S Midiri M Galluzzo A Group ftAS: Visceral Adiposity Index Diabetes Care 2010 33 920 922 10.2337/dc09-1825 20067971 PMC2845052

[B4] Bozorgmanesh M Hadaegh F Azizi F Diabetes prediction, lipid accumulation product, and adiposity measures; 6-year follow-up: Tehran lipid and glucose study Lipids Health Dis 2010 9 45 10.1186/1476-511X-9-45 20459710 PMC2876156

[B5] Bozorgmanesh M Hadaegh F Azizi F Predictive performances of lipid accumulation product vs. adiposity measures for cardiovascular diseases and all-cause mortality, 8.6-year follow-up: Tehran lipid and glucose study Lipids Health Dis 2010 9 100 10.1186/1476-511X-9-100 20846382 PMC2949857

[B6] Azizi F Ghanbarian A Momenan AA Hadaegh F Mirmiran P Hedayati M Mehrabi Y Zahedi-Asl S Prevention of non-communicable disease in a population in nutrition transition: Tehran Lipid and Glucose Study phase II Trials 2009 10 5 19166627 10.1186/1745-6215-10-5 PMC2656492

[B7] Freedman DS Kahn HS Mei Z Grummer-Strawn LM Dietz WH Srinivasan SR Berenson GS Relation of body mass index and waist-to-height ratio to cardiovascular disease risk factors in children and adolescents: the Bogalusa Heart Study Am J Clin Nutr 2007 86 33 40 17616760 10.1093/ajcn/86.1.33

[B8] Hadaegh F Harati H Ghanbarian A Azizi F Association of total cholesterol versus other serum lipid parameters with the short-term prediction of cardiovascular outcomes: Tehran Lipid and Glucose Study Eur J Cardiovasc Prev Rehabil 2006 13 571 577 10.1097/01.hjr.0000216552.81882.ca 16874147

[B9] Gibbons RJ Abrams J Chatterjee K Daley J Deedwania PC Douglas JS Ferguson TB Jr Fihn SD Fraker TD Jr Gardin JM ACC/AHA 2002 Guideline Update for the Management of Patients With Chronic Stable Angina-Summary Article: A Report of the American College of Cardiology/American Heart Association Task Force on Practice Guidelines (Committee on the Management of Patients With Chronic Stable Angina) Circulation 2003 107 149 158 10.1161/01.CIR.0000047041.66447.29 12515758

[B10] Braunwald E Antman EM Beasley JW Califf RM Cheitlin MD Hochman JS Jones RH Kereiakes D Kupersmith J Levin TN ACC/AHA Guideline Update for the Management of Patients With Unstable Angina and Non-ST-Segment Elevation Myocardial Infarction-2002: Summary Article: A Report of the American College of Cardiology/American Heart Association Task Force on Practice Guidelines (Committee on the Management of Patients With Unstable Angina) Circulation 2002 106 1893 1900 10.1161/01.CIR.0000037106.76139.53 12356647

[B11] Genuth S Alberti KG Bennett P Buse J Defronzo R Kahn R Kitzmiller J Knowler WC Lebovitz H Lernmark A Follow-up report on the diagnosis of diabetes mellitus Diabetes Care 2003 26 3160 3167 14578255 10.2337/diacare.26.11.3160

[B12] D'Agostino RB Sr Vasan RS Pencina MJ Wolf PA Cobain M Massaro JM Kannel WB General Cardiovascular Risk Profile for Use in Primary Care: The Framingham Heart Study Circulation 2008 117 743 753 10.1161/CIRCULATIONAHA.107.699579 18212285

[B13] Bozorgmanesh M Hadaegh F Azizi F Predictive accuracy of the 'Framingham's general CVD algorithm' in a Middle Eastern population: Tehran Lipid and Glucose Study International Journal of Clinical Practice 2011 65 264 273 10.1111/j.1742-1241.2010.02529.x 21314863

[B14] Abbate LM Stevens J Schwartz TA Renner JB Helmick CG Jordan JM Anthropometric measures, body composition, body fat distribution, and knee osteoarthritis in women Obesity (Silver Spring) 2006 14 1274 1281 10.1038/oby.2006.145 16899809

[B15] D'Agostino RB Nam BH Balakrishnan N, Rao CR Evaluation of the performance of survival analysis models: Discrimination and Calibration measures Handbook of Statistics, Survival Methods 2004 23 Amsterdam, The Netherlands: Elsevier B.V. 1 25

[B16] Hosmer DW Lemeshow S Applied logistic regression 2000 Wiley-Interscience

[B17] Hosmer DW Lemeshow S May S Applied survival analysis: regression modeling of time-to-event data 2008 2 Hoboken, N.J.: Wiley-Interscience

[B18] Hanley JA McNeil BJ The meaning and use of the area under a receiver operating characteristic (ROC) curve Radiology 1982 143 29 36 7063747 10.1148/radiology.143.1.7063747

[B19] Harrell FE Jr Lee KL Mark DB Multivariable prognostic models: issues in developing models, evaluating assumptions and adequacy, and measuring and reducing errors Stat Med 1996 15 361 387 10.1002/(SICI)1097-0258(19960229)15:4<361::AID-SIM168>3.0.CO;2-4 8668867

[B20] Hlatky MA Greenland P Arnett DK Ballantyne CM Criqui MH Elkind MSV Go AS Harrell FE Jr Hong Y Howard BV Criteria for Evaluation of Novel Markers of Cardiovascular Risk: A Scientific Statement From the American Heart Association Circulation 2009 119 2408 2416 10.1161/CIRCULATIONAHA.109.192278 19364974 PMC2956982

[B21] Pencina MJ D'Agostino RB Sr D'Agostino RB Jr Vasan RS Evaluating the added predictive ability of a new marker: from area under the ROC curve to reclassification and beyond Stat Med 2008 27 157 172 discussion 207-112 10.1002/sim.2929 17569110

[B22] Law MR Wald NJ Risk factor thresholds: their existence under scrutiny BMJ 2002 324 1570 1576 10.1136/bmj.324.7353.1570 12089098 PMC1123506

[B23] Rose G The strategy of preventive medicine Rose&# 39; Strategy of Preventive Medicine 2008 1 33 35

[B24] Amato MC Giordano C Pitrone M Galluzzo A Cut-off points of the visceral adiposity index (VAI) identifying a visceral adipose dysfunction associated with cardiometabolic risk in a Caucasian Sicilian population Lipids in Health and Disease 2011 10 183 10.1186/1476-511X-10-183 22011564 PMC3224548

[B25] Gelber RP Gaziano JM Orav EJ Manson JE Buring JE Kurth T Measures of Obesity and Cardiovascular Risk Among Men and Women J Am Coll Cardiol 2008 52 605 615 10.1016/j.jacc.2008.03.066 18702962 PMC2671389

[B26] Hermans MP Sacks FM Ahn SA Rousseau MF Non-HDL-cholesterol as valid surrogate to apolipoprotein B100 measurement in diabetes: Discriminant Ratio and unbiased equivalence Cardiovasc Diabetol 2011 10 20 10.1186/1475-2840-10-20 21356116 PMC3056766

[B27] Knowles KM Paiva LL Sanchez SE Revilla L Lopez T Yasuda MB Yanez ND Gelaye B Williams MA Waist circumference, body mass index, and other measures of adiposity in predicting cardiovascular disease risk factors among Peruvian adults 2011 Journal of Hypertension: International 2011 10.4061/2011/931402 PMC3034939 21331161

[B28] Dong X Liu Y Yang J Sun Y Chen L Efficiency of anthropometric indicators of obesity for identifying cardiovascular risk factors in a Chinese population Postgraduate Medical Journal10.1136/pgmj.2010.100453 21273363

[B29] Sacco RL Khatri M Rundek T Xu Q Gardener H Boden-Albala B Di Tullio MR Homma S Elkind MSV Paik MC Improving Global Vascular Risk Prediction With Behavioral and Anthropometric Factors: The Multiethnic NOMAS (Northern Manhattan Cohort Study) J Am Coll Cardiol 2009 54 2303 2311 10.1016/j.jacc.2009.07.047 19958966 PMC2812026

[B30] Schneider HJ Friedrich N Klotsche J Pieper L Nauck M John U Dorr M Felix S Lehnert H Pittrow D The Predictive Value of Different Measures of Obesity for Incident Cardiovascular Events and Mortality J Clin Endocrinol Metab 2010 95 1777 1785 10.1210/jc.2009-1584 20130075

[B31] Hadaegh F Zabetian A Sarbakhsh P Khalili D James WP Azizi F Appropriate cutoff values of anthropometric variables to predict cardiovascular outcomes: 7.6 years follow-up in an Iranian population Int J Obes (Lond) 2009 33 1437 1445 10.1038/ijo.2009.180 19752876

[B32] Despres JP Is visceral obesity the cause of the metabolic syndrome? Ann Med 2006 38 52 63 10.1080/07853890500383895 16448989

[B33] Lebovitz HE Banerji MA Point: Visceral Adiposity Is Causally Related to Insulin Resistance Diabetes Care 2005 28 2322 2325 10.2337/diacare.28.9.2322 16123512

[B34] Kramer CK von Muhlen D Gross JL Barrett-Connor E A Prospective Study of Abdominal Obesity and Coronary Artery Calcium Progression in Older Adults J Clin Endocrinol Metab 2009 94 5039 5044 10.1210/jc.2009-1497 19846732 PMC2795663

[B35] Gutin B Johnson MH Humphries MC Hatfield-Laube JL Kapuku GK Allison JD Gower BA Daniels SR Barbeau P Relationship of Visceral Adiposity to Cardiovascular Disease Risk Factors in Black and White Teens[ast] Obesity 2007 15 1029 1035 10.1038/oby.2007.602 17426339

[B36] Moebus S Balijepalli C Lösch C Göres L von Stritzky B Bramlage P Wasem J Jöckel KH Age-and sex-specific prevalence and ten-year risk for cardiovascular disease of all 16 risk factor combinations of the metabolic syndrome-A cross-sectional study Cardiovasc Diabetol 2010 9 34 10.1186/1475-2840-9-34 20696055 PMC2929217

